# Characteristics of the oral glucose tolerance test in women with different pre-pregnancy body mass index and the effect of gestational diabetes mellitus on twin pregnancy outcomes

**DOI:** 10.1016/j.clinsp.2023.100272

**Published:** 2023-08-19

**Authors:** Jinying Luo, Xiaoyan Geng, Jinfu Zhou, Shengnan Liang, Wei Zheng, Guanghui Li

**Affiliations:** a Division of Endocrinology and Metabolism, Department of Obstetrics, Beijing Obstetrics and Gynecology Hospital, Capital Medical University, PR China; b Beijing Maternal and Child Health Care Hospital, PR China; c Department of Obstetrics, Fujian Maternity and Child Health Hospital, College of Clinical Medicine for Obstetrics & Gynecology and Pediatrics, Fujian Medical University, PR China; d Department of Obstetrics and Gynecology, Xicheng Women and Children's Health Hospital, PR China

**Keywords:** Twin pregnancy, Gestational diabetes mellitus, Glucose tolerance test, Body mass index, Pregnancy outcomes

## Abstract

**Objective:**

This study aimed to investigate the mid-pregnancy blood glucose levels of women with singleton or twin pregnancies.

**Method:**

The relationship between blood glucose levels and Gestational Diabetes Mellitus (GDM) was studied in women with different pre-pregnancy Body Mass Index (BMI), and the effect of GDM on twin pregnancy outcomes was analyzed. Women with twin (*n* = 1,985) and singleton (*n* = 1,985) pregnancies were categorized into underweight (BMI < 18.5 kg/m^2^, *n* = 597), normal weight (BMI: 18.5–23.9 kg/m^2^, *n* = 2,575), and overweight/obese (BMI ≥ 24 kg/m^2^, *n* = 798) groups.

**Results:**

The incidence of GDM was 21.01% in women with twin pregnancies. Among the women with GDM in twin pregnancies, 38.37% had at least two abnormal blood glucose levels. The incidence of these parameters increased with preconception BMI, and the incidence of twin pregnancies was higher than that of singleton pregnancies (*p* < 0.001). In the normal weight and overweight/obese group, the oral glucose tolerance test glucose level and incidence of GDM were higher in women with twin than singleton pregnancies (*p* < 0.05). For twin pregnancies, the prevalence of selective fetal growth restriction was higher and anemia was lower in the GDM group than in the non-GDM group (all *p* < 0.05).

**Conclusion:**

Therefore, a greater emphasis should be placed on BMI before conception, and well-controlled GDM does not increase adverse pregnancy outcomes for twin pregnancies.

## Highlights

• The oral glucose tolerance characteristics were analyzed.
• Effect of gestational diabetes on the pregnancy outcome of twins was analyzed.
• The influence of preconception BMI on twin pregnancy outcome was explored.

## Introduction

The increasing sophistication and popularity of assisted reproductive technologies have led to an increase in the incidence of multiple pregnancies (especially twin pregnancies), with an incidence of up to 3%.^1^ Twin pregnancies are considered high-risk pregnancies and are more prone to complications, including preterm labor, gestational hypertension, and gestational diabetes mellitus (GDM).^2^ GDM can increase adverse pregnancy outcomes,^3^ is an early risk factor for maternal type 2 diabetes mellitus, cardiovascular disease, and impaired renal function, and adversely affects metabolism in children and adolescents.^4^, ^5^, ^6^ GDM is related to the age of pregnant women, pre-pregnancy weight, and weight gain during pregnancy, among other factors.^7^ It may be expected that the degree of insulin resistance and consequently the rate of GDM would be higher in women with twin pregnancies. However, the presence of two fetuses and higher maternal basal metabolic rate may be associated with increased utilization of glucose, which could counteract the increased insulin resistance to some degree.

Previous research on GDM has mainly focused on singleton pregnancies and have established the relationship between GDM and pre-pregnancy weight and perinatal outcomes.^3^, ^4^, ^5^, ^6^, ^7^ Whether this relationship also applies to twin pregnancies is yet to be elucidated. To the best of our knowledge, no large-scale studies have been reported on the relationship between blood glucose levels and GDM and pre-pregnancy weight in patients with twin pregnancies. There is also a lack of consistent conclusions about whether GDM increases adverse outcomes in twin pregnancies.

Therefore, this study aimed to investigate the differences in blood glucose levels measured using the 75 g oral glucose tolerance test (OGTT) and GDM incidence between women with singleton pregnancies and those with twin pregnancies. The relationship between GDM and pre-pregnancy body mass index (BMI) was determined, and the effect of GDM on twin pregnancy outcomes was also analyzed.

## Materials and methods

### Data sources

The clinical data of 1985 patients with twin pregnancies and 1985 patients with singleton pregnancies who underwent the 75 g OGTT and delivered at the Beijing Obstetrics and Gynecology Hospital or Fujian Maternity and Child Health, which process > 15,000 annual deliveries, between May 1, 2015, and June 30, 2018, were analyzed. Maternal age, height, pre-pregnancy body weight, pre-pregnancy BMI, and 75 g OGTT results were collected from the medical charts. Patients who did not undergo the 75 g OGTT and those with missing pre-pregnancy BMI or height data, pre-GDM, or triplet or higher order pregnancies were excluded from the study. This study was conducted in accordance with the Declaration of Helsinki, was approved by the institutional review boards of the participating institutions (protocol code: 2018-ky-009–01 and 2020KY117), and follows the STROBE Statement.

### Methods

#### Diagnosis of GDM

GDM was diagnosed according to the methods and criteria stated in the Guidelines for the Diagnosis and Treatment of Pregnancy with Diabetes (2014 edition),^8^ and the classification criteria based on pre-pregnancy BMI stated in the China Expert Consensus on Medical Nutrition Therapy for Overweight and Obesity (2016 edition)^9^ were used to group patients based on BMI.

Patients were instructed to consume at least 150 g of carbohydrates per day for 3 consecutive days prior to OGTT. On the day of the test, the patients fasted for at least eight hours before the test and were asked to rest and refrain from smoking during the test. Each patient was instructed to drink a 300 mL solution containing 75 g of glucose within 5 min. Venous blood was drawn prior to glucose ingestion (fasting blood glucose, OGTT0h blood glucose) and one (OGTT1h blood glucose) and two (OGTT2h blood glucose) hours after the start of glucose ingestion. The glucose oxidase method was used to determine the blood glucose levels.

A fasting blood glucose ≥ 5.1 mmoL/L (92 mg/dL), an OGTT1h blood glucose ≥ 10.0 mmoL/L (180 mg/dL), and an OGTT2h blood glucose ≥ 8.5 mmoL/L (153 mg/dL) were considered abnormal. Patients with at least one abnormal blood glucose value were diagnosed with GDM.

#### Grouping

Patients who were pregnant with singletons and twins were grouped based on their pre-pregnancy BMI into an underweight group (BMI < 18.5 kg/m^2^, *n* = 597), including 288 women with singleton and 309 with twin pregnancies; normal weight group (BMI: 18.5–23.9 kg/m^2^, *n* = 2575), including 1304 women with singleton and 1271 with twin pregnancies; and overweight/obese group (BMI ≥ 24 kg/m^2^, *n* = 798), including 393 women with singleton and 405 with twin pregnancies.

#### Body weight measurement

A digital scale was used to measure each patient's body weight. Body weight data recorded within one month prior to conception were used as the pre-pregnancy body weight values.

#### Main pregnancy outcomes of twin pregnancies

Major pregnancy outcomes included pregnancy-induced hypertension, preeclampsia, premature rupture of membranes, postpartum hemorrhage, preterm birth, anemia, Twin to Twin Transfusion Syndrome (TTTS), Selective Fetal Growth Restriction (SFGR), Small for Gestational Age (SGA), admission of any infant to the Neonatal Intensive Care Unit (NICU), and asphyxia of any infant.

#### Indicators

The differences in blood glucose levels and the incidence of GDM between singleton and twin pregnant women were analyzed.

Fasting, OGTT1h and OGTT2h blood glucose levels in the pre-pregnancy BMI group were compared to determine the relationship between pre-pregnancy BMI and the incidence of GDM.

The effect of GDM on twin pregnancy outcomes was analyzed.

### Statistical analyses

Continuous data are presented as mean ± standard deviation or median (range). The composition ratio and rate are expressed as frequencies (%). The *t*-test, Chi-Square test, and one-way analysis of variance were used to determine differences between the groups. All statistical analyses were performed using SPSS version 17.0 (SPSS Inc. Released 2008. SPSS Statistics for Windows, Version 17.0. Chicago: SPSS Inc.). Statistical significance was set at *p* < 0.05.

## Results

### Baseline characteristics

A total of 1985 women were enrolled in the singleton and twin pregnancy groups. Among them, 845 singleton pregnancies and 1154 twin pregnancies were from Beijing Obstetrics and Gynecology Hospital, and 1140 singleton pregnancies and 831 twin pregnancies were from Fujian Maternity and Child Health Hospital. There were no significant differences in mean age, mean pre-pregnancy BMI and OGTT test time among groups (*p* > 0.05). The proportion of assisted reproduction in twin pregnancies was higher than that in singleton pregnancies (*p* < 0.05). Baseline data from the patients are reported in Table 1.

**Table 1:** Comparison of baseline data of singleton and twin pregnancies.

Index	Singleton pregnancies	Twin pregnancies	T	p-value
Age (y)	31.10 ± 4.05	31.15 ± 4.20	0.412	0.680
Height (m)	1.61 ± 0.05	1.62 ± 0.05	3.444	0.001
Pre-pregnancy weight (kg)	55.92 ± 8.57	56.50 ± 8.78	2.129	0.033
Pre-pregnancy BMI (kg/m^2^)	21.53 ± 2.98	21.60 ± 3.11	0.751	0.452
Assisted reproductive conception (%)	3.68 (73/1985)	44.73 (888/1985)	911.927	0.000
OGTT test time (week)	25±0.25	25.5 ± 0.12	0.413	0.681

BMI, Body Mass Index; OGTT, Oral Glucose Tolerance Test.

Overall, women with twin pregnancies had significantly higher OGTT outcomes at three-time points than women with singleton pregnancies (*p* < 0.001) (Table 2). The incidence of GDM in twin pregnancies was 21.01%, which was significantly higher than that in singleton pregnancies (16.57%) (*p* < 0.001). The incidence of GDM was 20.80% (240/1154) in Beijing Maternal and Child Health Care Hospital and 21.30% (177/831) in Fujian Maternity and Child Health Hospital. The incidence of GDM in women with singleton pregnancy was higher in Fujian Maternity and Child Health Hospital 14.56% (166/1140) than in Beijing Maternal and Child Health Care Hospital 19.29% (163/845). The proportion of GDM with ≥ 2 abnormalities in twin pregnancy (38.37%) was significantly higher than that in singleton pregnancy (25.84%) (*p* < 0.05). The proportion of GDM with one abnormality in the twin pregnancy group (61.63%) was significantly lower than that in the singleton pregnancy group (74.16%) (*p* < 0.05) (Table 2).

**Table 2:** Comparison of blood glucose related indexes in singleton and twin pregnancy.

Index	Singleton pregnancies	Twin pregnancies	T	p-value
Fasting (mmoL/L)	4.47 ± 0.43	4.54 ± 0.46	5.264	0.000
OGTT1h (mmoL/L)	7.97 ± 1.63	8.21 ± 1.62	4.629	0.000
OGTT2h (mmoL/L)	6.47 ± 1.25	6.78 ± 1.33	7.727	0.000
Incidence of GDM (%)	16.57 (329/1985)	21.01 (417/1985)	12.783	0.000
Constituent ratio of only one abnormal blood glucose value (%)	74.16 (244/329)	61.63 (257/417)	13.098	0.000
Constituent ratio of at least two abnormal blood glucose value (%)	25.84 (85/329)	38.37 (160/417)	13.098	0.000
Constituent ratio of only fasting abnormal blood glucose value (%)	23.10 (76/329)	23.02 (96/417)	0.001	0.000
Constituent ratio of only OGTT1h abnormal blood glucose value (%)	37.69 (124/329)	18.47 (77/417)	34.530	0.000
Constituent ratio of only OGTT2h abnormal blood glucose value (%)	13.37 (44/329)	20.14 (84/417)	5.930	0.000

OGTT, Oral Glucose Tolerance Test; GDM, Gestational Diabetes Mellitus.

### Comparison of blood glucose levels and the incidence of GDM in pregnant women with different pre-pregnancy BMI

According to pre-pregnancy BMI, there were 597 patients in the underweight group, including 288 with singleton and 309 with twin pregnancies; 2575 patients in the normal weight group, including 1304 with singleton and 1271 with twin pregnancies; and 798 patients in the overweight/obese group, including 393 with singleton and 405 with twin pregnancies.

As pre-pregnancy BMI increased, fasting, OGTT1h, and OGTT2h blood glucose levels also increased in both groups (*p* < 0.001). However, these variables were significantly higher in women with twin pregnancies than in those with singleton pregnancies (*p* < 0.001) (Table 3).

**Table 3:** Comparison of blood glucose related indexes in pregnant women with different pre-pregnancy BMI OGTT, oral.

BMI group	Fasting (mmoL/L)		OGTT1h (mmoL/L)		OGTT2h (mmoL/L)	
	Singleton	Twin	Singleton	Twin	Singleton	Twin
Underweight	4.40 ± 0.37	4.47 ± 0.42	7.60 ± 1.54	7.87 ± 1.58	6.31 ± 1.19	6.41 ± 1.31
Normal weight	4.45 ± 0.41	4.51 ± 0.42	7.92 ± 1.61	8.12 ± 1.55	6.44 ± 1.23	6.80 ± 1.31
Overweight/obese	4.60 ± 0.53	4.72 ± 0.56	8.41 ± 1.66	8.74 ± 1.75	6.66 ± 1.35	7.00 ± 1.36
F	23.878	41.589	22.748	31.662	7.092	18.386
p-value	0.000	0.000	0.000	0.000	0.000	0.000

BMI, Body Mass Index; OGTT, Oral Glucose Tolerance Test. Data are reported as the mean ± standard deviation.

There were significant differences in the incidence of GDM, abnormal proportion of 1 GDM marker and abnormal proportion of at least 2 GDM markers between singleton pregnancy and twin pregnancy (*p* < 0.05). As BMI increased, the incidence of GDM, the proportion of at least two GDM abnormalities, and the incidence of elevated fasting blood glucose (FAsted) increased in both singleton and twin pregnancies. The proportion of women with one type of GDM abnormality was highest in the normal-weight group (Table 4).

**Table 4:** Comparison of blood glucose related indexes among groups of pregnant women with different pre-pregnancy BMI in singleton and twin pregnancies (continued).

BMI group	Incidence of GDM (%)		Constituent ratio of only one abnormal blood glucose value (%)		Constituent ratio of at least two abnormal blood glucose values (%)		Constituent ratio of only fasting abnormal blood glucose value (%)		Constituent ratio of only OGTT1h abnormal blood glucose value (%)		Constituent ratio of only OGTT2h abnormal blood glucose value (%)	
	Singleton	Twin	Singleton	Twin	Singleton	Twin	Singleton	Twin	Singleton	Twin	Singleton	Twin
Underweight	11.46 (33/288)	14.24 (44/309)	9.38 (27/288)	8.74 (27/309)	2.08 (6/288)	5.50 (17/309)	3.82 (11/288)	3.56 (11/309)	4.17 (12/288)	2.27 (7/309)	1.39 (4/288)	2.91 (9/309)
Normal weight	14.65 (191/1304)	19.35 (246/1271)	11.81 (154/1304)	12.98 (165/1271)	2.84 (37/1304)	6.37 (81/1271)	2.84 (37/1304)	4.48 (57/1271)	6.83 (89/1304)	3.86 (49/1271)	2.15 (28/1304)	4.64 (59/1271)
Overweight/ Obese	26.72 (105/393)	32.32 (127/393)	7.89 (63/798)	16.54 (65/393)	5.26 (42/798)	15.78 (62/393)	3.51 (28/798)	7.12 (28/393)	2.88 (23/798)	5.34 (21/393)	1.50 (12/798)	4.07 (16/393)
F	38.196	36.768	7.649	8.317	49.371	36.325	15.075	5.233	3.155	4.013	2.209	1.934
p-value	0.000	0.000	0.022	0.016	0.000	0.000	0.001	0.073	0.206	0.134	0.331	0.380

BMI, Body Mass Index; OGTT, Oral Glucose Tolerance Test.

In the underweight group, the fasting and OGTT1h blood glucose levels of women with twin pregnancies were higher than those of women with singleton pregnancies (*p* < 0.05), but there were no statistically significant differences for other indices (*p* > 0.05) (Table 5).

**Table 5:** Comparison of blood glucose-related indices in pregnant women with different pre-pregnancy BMI.

Underweight group	Singleton (*n* = 288)	Twin (*n* = 309)	T	p-value
Fasting (mmoL/L)	4.40 ± 0.37	4.47 ± 0.42	1.977	0.049
OGTT1h (mmoL/L)	7.60 ± 1.54	7.87 ± 1.58	2.084	0.038
OGTT2h (mmoL/L)	6.31 ± 1.19	6.41 ± 1.31	0.905	0.364
Incidence of GDM (%)	11.46 (33/288)	14.24 (44/309)	1.026	0.311
Constituent ratio of only one abnormal blood glucose value (%)	81.82 (27/33)	61.36 (27/44)	0.074	0.786
Constituent ratio of at least two abnormal blood glucose values (%)	18.18 (6/33)	38.64 (17/44)	4.702	0.030
Constituent ratio of only fasting abnormal blood glucose value (%)	3.82 (11/288)	3.56 (11/309)	0.028	0.866
Constituent ratio of only OGTT1h abnormal blood glucose value (%)	4.17 (12/288)	2.27 (7/309)	1.749	0.186
Constituent ratio of only OGTT2h abnormal blood glucose value (%)	1.39 (4/288)	2.91 (9/309)	1.625	0.202
Comparison of blood glucose-related indices in pregnant women with different pre-pregnancy BMI (continued).				


Normal weight group	Singleton (*n* = 1304)	Twin (*n* = 1271)	T	p-value
Fasting (mmoL/L)	4.45 ± 0.41	4.51 ± 0.42	3.703	0.000
OGTT1h (mmoL/L)	7.92 ± 1.61	8.12 ± 1.55	3.2	0.001
OGTT2h (mmoL/L)	6.44 ± 1.23	6.80 ± 1.32	7.186	0.000
Incidence of GDM (%)	15.16 (191/1304)	19.63 (246/1271)	10.12	0.001
Constituent ratio of only one abnormal blood glucose value (%)	80.63 (154/191)	67.07 (165/246)	0.815	0.367
Constituent ratio of at least two abnormal blood glucose values (%)	19.37 (37/191)	32.93 (81/246)	18.4	0.000
Constituent ratio of only fasting abnormal blood glucose value (%)	10.56 (37/1304)	16.55 (57/1271)	4.965	0.026
Constituent ratio of only OGTT1h abnormal blood glucose value (%)	41.67 (90/1304)	22.30 (49/1271)	11.70	0.001
Constituent ratio of only OGTT2h abnormal blood glucose value (%)	18.52 (28/1304)	28.06 (59/1271)	12.27	0.000
Comparison of blood glucose related indices in pregnant women with different pre-pregnancy BMI (continued).				

Overweight/obese group	Singleton (*n* = 393)	Twin (*n* = 405)	T	p-value
Fasting (mmoL/L)	4.60 ± 0.53	4.72 ± 0.56	3.234	0.001
OGTT1h (mmoL/L)	8.41 ± 1.66	8.74 ± 1.75	2.780	0.006
OGTT2h (mmoL/L)	6.66 ± 1.35	7.01 ± 1.36	3.608	0.000
Incidence of GDM (%)	26.72 (105/393)	31.36 (127/405)	4.000	0.030
Constituent ratio of only one abnormal blood glucose value (%)	16.03 (63/393)	16.05 (65/405)	0.003	0.957
Constituent ratio of at least two abnormal blood glucose values (%)	10.69 (42/393)	15.31 (62/405)	3.551	0.059
Constituent ratio of only fasting abnormal blood glucose value (%)	7.12 (28/393)	6.91 (28/405)	0.024	0.887
Constituent ratio of only OGTT1h abnormal blood glucose value (%)	5.85 (23/393)	5.19 (21/405)	0.199	0.655
Constituent ratio of only OGTT2h abnormal blood glucose value (%)	3.05 (12/393)	3.95 (16/405)	0.438	0.508

BMI, Body Mass Index; OGTT, Oral Glucose Tolerance Test; GDM, Gestational Diabetes Mellitus.

In the normal weight group, the fasting, OGTT1h, and OGTT2h blood glucose levels, incidence of GDM, and constituent ratio of at least two abnormal blood glucose values were higher in women with twin pregnancies than in women with singleton pregnancies (*p* < 0.05) (Table 5 continued).

In the overweight/obese group, the fasting, OGTT1h, and OGTT2h blood glucose levels and the incidence of GDM were significantly higher in women with twin pregnancies than in those with singleton pregnancies (*p* < 0.05). There were no statistically significant differences in other indices (*p* > 0.05) (Table 5 continued).

### Effect of GDM on pregnancy outcomes in women with twin pregnancies

Women with GDM and twin pregnancies were consulted at a nutrition clinic and received standardized blood glucose monitoring and treatment; the overall blood glucose control was good. The sFGR was higher and anemia prevalence was lower in the GDM group than in the non-GDM group (*p* < 0.05). However, there were no significant differences between GDM and non-GDM groups in other pregnancy outcomes, such as gestational hypertension, preeclampsia, premature rupture of membranes, TTTS, postpartum hemorrhage, preterm labor, SGA, NICU admission, and neonatal asphyxia (*p* > 0.05) (Table 6).

**Table 6:** Comparison of major pregnancy outcomes between women with twin pregnancies with and without GDM.

Group	GDM (*n* = 417)	Non-GDM (*n* = 1568)	X^2^	p-value
gestational hypertension	5.28 (22/417)	4.15 (65/1568)	1.004	0.316
preeclampsia	11.99 (50/417)	13.01 (204/1568)	0.307	0.580
Premature rupture of membranes	17.75 (74/417)	18.69 (293/1568)	0.193	0.660
Postpartum hemorrhage	9.35 (39/417)	10.08 (158/1568)	0.193	0.660
Preterm labor	45.80 (191/417)	45.85 (719/1568)	0.000	0.985
Anemia	33.09 (138/417)	40.75 (639/1568)	8.112	0.004
TTTS	1.68 (7/417)	1.98 (31/1568)	0.156	0.693
SFGR	3.60 (15/417)	1.59 (25/1568)	6.691	0.010
SGA of any one baby	29.98 (125/417)	30.61 (480/1568)	0.063	0.802
NICU admission of any one baby	24.22 (101/417)	24.04 (377/1568)	0.006	0.940
Neonatal asphyxia of any one baby	0.24 (1/417)	0.45 (7/1568)	/	1.000

GDM, Gestational Diabetes Mellitus; SGA, Small for Gestational Age; NICU, Neonatal Intensive Care Unit.

## Discussion

GDM is the most common endocrine and metabolic disorder in pregnancy, affecting both mother and fetus. Therefore, it is of great clinical significance to study the blood glucose level of pregnant women with gestational diabetes. In this study, blood glucose levels of patients with twin pregnancies (a group at high risk for GDM) were compared with those in patients with singleton pregnancies. Patients with twin pregnancies were diagnosed with GDM more frequently and had a higher number of abnormal blood glucose values than those with singleton pregnancies. Furthermore, the number of abnormal blood glucose values was associated with pre-pregnancy BMI. However, GDM in women with twin pregnancies did not significantly increase maternal adverse pregnancy outcomes.

### Differences in blood glucose levels and incidence of GDM between patients with singleton and twin pregnancies

The simultaneous growth of two fetuses may cause a greater degree of endocrine and metabolic changes, increasing the possibility of glucose metabolism disorders.^10^ The current study has shown that fasting, OGTT1h, and OGTT2h blood glucose levels in women with twin pregnancies are higher than in those with singleton pregnancies, suggesting blood glucose levels during the second trimester of twin pregnancy is higher than that in a singleton pregnancy. In terms of the overall incidence of GDM, the incidence of GDM in 1985 cases of twin pregnancy included in this study was 21.01%, which is close to the 20.4% reported in a previous study conducted in China.^11^ Moreover, women with twin pregnancies were included from two hospitals with more than 15,000 annual deliveries in North and South China for comparison. It was found that the incidence of GDM in women with twin pregnancies was comparable in the north and south hospitals (20.80% vs. 21.30%), suggesting that the overall incidence of GDM is similar in maternal and child healthcare hospitals with large delivery volumes.

The incidence of GDM in women with twin pregnancies was reported to be 8.4%–14.7%, and was as high as 26% in 2013 in countries outside of China.^12^,^13^ For the incidence of GDM in singleton pregnant women, there are differences in domestic reports under the premise of adopting the same diagnostic criteria. A multicenter study involving 13 hospitals in China (including multiple pregnancies) showed an incidence of GDM of 17.5% (3002/17,186).^14^ But the incidence of GDM varies a large rank in different areas of China, which was demonstrated a GDM incidence of 7.1% in Hebei Province,^15^ while 21.00% of Ningbo City in Zhejiang Province and 21.76% of Peking City.^16^,^17^ It is speculated that the possible reasons involve selection bias arising from single-center research. Further, compared with general hospitals, the total number of births in maternity and child health hospitals is large, and the majority of pregnant women have low-risk pregnancies, which makes the overall incidence of GDM low.

Previous studies have also reported mixed results on whether there is a difference in the incidence of GDM among women with single and twin pregnancies. Studies conducted in the 1980s found no significant difference in the incidence of GDM among women with single and twin pregnancies.^18^, ^19^, ^20^, ^21^ With the constant change of diagnostic criteria and the deepening of GDM research, differences between pregnancy types have gradually been discovered. Most researchers have documented that twin pregnancies present a higher risk than singleton pregnancies.^12^,^22^,^23^ For instance, a case-control study conducted in Canada based on a large sample (3901 twin and 266,942 singleton pregnancies) showed that the overall incidence of GDM in women with twin pregnancies was higher than that in women with singleton pregnancies (8.4% vs. 6.3%, *p* < 0.001). Moreover, “light GDM”, which can be simply controlled through the diet, is dominant.^12^

Given the differences between singleton and twin pregnancies, some scholars believe that the screening method for GDM in women with twin pregnancies should use a different cut-off value; specifically, a cut-off value of 135 mg/dL for 50 g of glucose rather than 100 mg/dL for singleton pregnancies.^24^ In terms of the long-term effects, a study from Canada found that, for any given 75 g OGTT value, women with twin pregnancies are less likely to develop maternal type 2 diabetes mellitus in the future compared to women with singleton pregnancies. Based on the risk of future maternal type 2 diabetes mellitus, the threshold for diagnosis of GDM using an OGTT based on twin pregnancy-specific results was established as follows: 5.8 mmoL/L (104 mg/dL), 11.8 mmoL/L (213 mg/dL), and 10.4 mmoL/L (187 mg/dL).^25^ However, these thresholds have not been widely used in clinical practice. Relative to singleton pregnancies, twin pregnancies may be associated with increased placental mass, levels of some placental hormones (such as human placental lactogenogen and steroid hormones), levels of insulin resistance, and incidence of GDM. Since the incidence of GDM in women with twin pregnancies is close to that of women with singleton pregnancies at the Peking University First Hospital (21.1%, 1290/6013),^17^ the relevance of pregnancy type still needs to be further demonstrated in multi-center, multi-region studies involving different types of healthcare institutions and larger samples of women both with singleton and twin pregnancies.

### Relationship between pre-pregnancy weight and GDM in women with singleton and twin pregnancies

The international diagnostic criteria for overweight and obesity are mostly recommended by the 2009 IOM (pre-pregnancy BMI of 25.0–29.9 and ≥ 30.0 kg/m^2^).^26^,^27^ Due to ethnicity-related differences in BMI, in China at present, most studies used modified BMI classification (pre-pregnancy BMI of 24.0–27.9 and ≥ 28 kg/m^2^ as the diagnostic criteria of overweight and obese, respectively). Domestic studies and those conducted outside of China have found that GDM is related to many factors, such as the age of pregnant women and pre-pregnancy BMI.^16^,^17^,^27^ Pre-pregnancy overweight or obesity is a high-risk factor for GDM,^20^ while twin pregnancies and singleton pregnancies have similar high-risk factors, among which obesity is an independent risk factor for GDM in women with twin pregnancies.^12^,^28^,^29^

The present study showed that OGTT blood glucose values at all three-time points tended to increase with increasing pre-pregnancy Body Mass Index (BMI). In addition, OGTT blood glucose values at all three-time points were higher in twin pregnant women in the normal weight group and overweight/obese group than in singleton pregnant women. Therefore, pre-pregnancy weight may affect the difference in blood glucose levels during the second trimester both in women with singleton and twin pregnancies. The present study also emphasized that the overall incidence of GDM in women with singleton and twin pregnancies tended to increase with increasing pre-pregnancy BMI, suggesting a positive correlation between pre-pregnancy BMI and the incidence of GDM, which further confirms the relationship between overweight or obesity and GDM. The comparison of women with singleton and twin pregnancies in different pre-pregnancy BMI groups showed that the incidence of GDM and blood glucose at three-time points of women with twin pregnancies in the normal weight and overweight/obese groups were higher than those of women with singleton pregnancies, which also reveals the influence of pre-pregnancy weight on blood glucose regulation. It is speculated that the main reasons for such are as follows: weight gain leads to decreased function of pancreatic β cells and increased insulin resistance,^30^ coupled with increased placental hormones resulting from increased placental mass during twin pregnancy which aggravates insulin resistance, and this double effect leads to increased incidence of GDM in women with twin pregnancies. In contrast to the present outcomes, a study conducted in Israel found that, although obesity can cause GDM in both women with singleton and twin pregnancies, the disease severity is less in women with twin compared to singleton pregnancies; however, the specific causes and mechanisms remain unclear. ^31^

In addition, the current diagnostic criteria for GDM are not as strict as in the past, with only one abnormality required for diagnosis. However, GDM with more than two abnormalities tends to be more serious and has a greater impact on the mother and child.^17^ Therefore, it is of greater clinical value to explore the correlation between more than two abnormalities of GDM and pre-pregnancy weight. The results of this study showed that as pre-pregnancy BMI increases, the composition ratio of more than two GDM abnormalities shows an increasing trend, which was more obvious in women with twin pregnancies, suggesting that pre-pregnancy overweight/obesity can aggravate GDM. The comparison of women with singleton and twin pregnancies with different preconception BMI showed that the composition ratio of more than two abnormalities among the three groups was higher in women with twin pregnancies than in those with singleton pregnancies, suggesting that twin pregnancy may aggravate the disease of GDM overall.

### Effect of GDM on pregnancy outcomes in women with twin pregnancies

A systematic review and meta-analysis of maternal and infant outcomes for GDM combined with singleton or twin pregnancies showed a significant increase in maternal outcomes (e.g., cesarean section) and neonatal outcomes (e.g., SGA, preterm birth, respiratory disease, hyperbilirubinemia, and NICU admissions) for GDM combined with twin pregnancies.^32^ Further, women with twin pregnancies and GDM were more likely to be older, of Hispanic or Asian race and ethnicity, married, obese, and have a college education and private insurance. After adjusting for potential confounding variables, patients with GDM were more likely to have hypertensive disease (18.0% vs. 10.2%) and to undergo cesarean section (51.2% vs. 47.3%) than patients without GDM. Newborns of patients with GDM were more likely to require > 6 h of mechanical ventilation (6.5% vs. 5.6%) and to be hospitalized in the NICU (41.1% vs. 36.2%), but less likely to have a lower birth weight or to be SGA (16.2% vs. 19.5%) compared to newborns of patients without GDM.^33^ Previous studies on the effect of GDM on twin pregnancy outcomes have found that GDM in women with twin pregnancies may increase the rate of cesarean section, premature delivery, and the occurrence of neonatal jaundice, but has no significant correlation with other pregnancy outcomes, such as hypertension during pregnancy, preeclampsia, neonatal respiratory diseases, NICU hospitalization, and neonatal hypoglycemia.^12^ Other studies have shown that GDM is not associated with adverse perinatal twin pregnancy outcomes; however, it may be positively correlated with hypertension in monochorionic diamniotic twins and negatively correlated with SGA (OR 2.68 and 0.35, respectively).^34^

Domestic and foreign studies have shown that GDM is not associated with adverse perinatal outcomes (such as preterm birth, “babies large for gestational age” and neonatal respiratory distress) in women with twin pregnancies after standardized management of GDM.^35^,^36^ Moreover, compared with the effect on singleton pregnancy outcomes, the effect of GDM on twin pregnancy outcomes was somewhat weakened.^37^ GDM appears to have a protective effect on the occurrence of SGA neonates in twin pregnancies.^38^ The results of this study showed that GDM had a certain effect on sFGR in twin pregnancies and a protective effect on anemia during pregnancy, but no effect was found on gestational hypertension, preeclampsia, premature rupture of membranes, TTTS, postpartum hemorrhage, premature labor, neonatal SGA, NICU admission, and neonatal asphyxia. Overall, well-controlled GDM had little effect on twin pregnancy outcomes.

In conclusion, the mid-pregnancy blood glucose level of twin pregnancies is higher than those of singleton pregnancies, and the incidence and severity of GDM are also higher. Therefore, women with twin pregnancies are a high-risk group for GDM, which is related to pre-pregnancy body weight, such that the risk of GDM is highest for overweight or obese women. Accordingly, it is recommended to maintain a healthy weight before pregnancy. Women with twin pregnancies should be screened for fasting blood glucose as soon as possible, and intervention measures, such as diet and exercise should be taken to reduce the occurrence of GDM and its adverse effects on mother and child.

There are still some limitations in this study. First, this is a retrospective study, which may have retrospective bias. Secondly, the patients were all from maternal and child health care hospitals, with mild GDM in general and few severe GDM patients. Therefore, the relationship between blood glucose in the second trimester and pre-pregnancy BMI should be further explored in the future by including critically ill patients in hospitals.

## Authors' contributions

Conceptualization, Jinying Luo and Guanghui Li; Data curation, Jinying Luo; Formal analysis, Jinying Luo and Wei Zheng; Funding acquisition, Jinying Luo and Guanghui Li; Investigation, Jinying Luo, Xiaoyan Geng and Shengnan Liang; Methodology, Jinying Luo; Project administration, Jinying Luo and Guanghui Li; Resources, Jinying Luo, Xiaoyan Geng and Shengnan Liang; Software, Jinfu Zhou; Supervision, Wei Zheng and Guanghui Li; Validation, Jinying Luo and Guanghui Li; Visualization, Wei Zheng and Guanghui Li; Writing – original draft, Jinying Luo; Writing – review & editing, Wei Zheng and Guanghui Li. All authors have read and approved the published version of the manuscript.

## Funding

This research was funded by the Beijing Hospitals Authority's Ascent Plan (grant number: DFL20191402) and Fujian Provincial Health Commission Young and Middle-aged Foundation Project (grant number: 2020GGB044).

## Declaration of Competing Interest

The authors declare no conflicts of interest.
